# Mobile phones improve case detection and management of malaria in rural Bangladesh

**DOI:** 10.1186/1475-2875-12-48

**Published:** 2013-02-04

**Authors:** Chai S Prue, Kerry L Shannon, Jacob Khyang, Laura J Edwards, Sabeena Ahmed, Malathi Ram, Timothy Shields, Mohammad S Hossain, Gregory E Glass, Myaing M Nyunt, David A Sack, David J Sullivan, Wasif A Khan

**Affiliations:** 1 International Centre for Diarrhoeal Disease Research, Dhaka, Bangladesh; 2 The Johns Hopkins Bloomberg School of Public Health, Baltimore, Maryland, USA; 3 The Johns Hopkins School of Medicine, Baltimore, Maryland, USA; 4 Furman University, Greenville, South Carolina, USA

**Keywords:** Mobile phones, Malaria, Bangladesh, Rapid test, Artemisinin, Surveillance, Rapid diagnostic test, mHealth

## Abstract

**Background:**

The recent introduction of mobile phones into the rural Bandarban district of Bangladesh provided a resource to improve case detection and treatment of patients with malaria.

**Methods:**

During studies to define the epidemiology of malaria in villages in south-eastern Bangladesh, an area with hypoendemic malaria, the project recorded 986 mobile phone calls from families because of illness suspected to be malaria between June 2010 and June 2012.

**Results:**

Based on phone calls, field workers visited the homes with ill persons, and collected blood samples for malaria on 1,046 people. 265 (25%) of the patients tested were positive for malaria. Of the 509 symptomatic malaria cases diagnosed during this study period, 265 (52%) were detected because of an initial mobile phone call.

**Conclusion:**

Mobile phone technology was found to be an efficient and effective method for rapidly detecting and treating patients with malaria in this remote area. This technology, when combined with local knowledge and field support, may be applicable to other hard-to-reach areas to improve malaria control.

## Background

Bangladesh is one of 106 countries endemic for malaria [1]. Most cases occur in 13 districts of the country, and the highest rates are seen in districts bordering Myanmar [2]. This hilly, forested area, known as the Chittagong Hill Tracts, is considered hypoendemic for malaria [3]. About 90% of the malarial infections are due to *Plasmodium falciparum*, all of which are chloroquine resistant. The rest of the malaria cases are diagnosed *Plasmodium vivax* with less than 1% *Plasmodium malariae*. Based on government reports, between 2008 and 2011, malaria cases decreased from 84,690 to 51,773 and malaria deaths decreased from 154 to 36, coincident with infusion of Global Fund money with distribution of bed nets, availability of rapid diagnostic tests (RDT) and artemisinin combination therapy (ACT) [4]. However, these reports may underestimate the true disease burden [2]. A cross sectional survey performed in 2007 as part of a nationwide survey estimated a prevalence of greater than 15% in Bandarban district of the Chittagong Hill Tracts [5].

The Government of Bangladesh, in collaboration with non-governmental organizations (NGOs) including BRAC and its partners, is implementing a malaria control programme in these 13 endemic districts with support from the Global Fund for AIDS, TB and Malaria starting in 2006. The programme focuses on early diagnosis of uncomplicated *P. falciparum* malaria using RDT, improved microscopy, and treatment with ACT (artemether-lumefantrine). They also emphasize prevention programmes by providing and promoting the use of long-lasting insecticide-treated nets. At the community level, health workers from both the government and NGOs are providing diagnostic and prevention services, distributing bed nets, and educating the community about strategies for malaria prevention.

To better understand the epidemiology of malaria in the Chittagong Hill Tracts of Bangladesh, the International Centre for Diarrhoeal Disease Research, Bangladesh (icddr, b) in collaboration with The Johns Hopkins Malaria Institute (JHMRI) initiated a study in 2009 called, “Mapping Malaria Epidemiology in Bangladesh” in two unions (Rajbilla and Kuhalong) of Bandarban sub district of the Chittagong Hill Tracts [3].

Since the project began in 2009, mobile phone access and use has increased dramatically in this field site, as it has in many remote areas of developing countries. The increased availability and use of mobile phones has the potential to improve health services [6]. They have been used in a variety of health applications such as reminding and encouraging the use of appropriate guidelines by healthcare workers and as a way to request assistance for maternal emergencies [7]. Mobile phones have also been used to facilitate care of patients with diabetes, HIV-AIDS, and asthma [8-10]. A few studies have reported on benefits of mobile phone use for malaria control in Africa and Asia [11-18]. Zurovac *et al.* note that mobile phones can play a vital role in malarial control by increasing communication both between patients and healthcare workers with respect to medication adherence and treatment review as well as between healthcare workers and service managers for disease surveillance, commodity monitoring, pharmocovigilance, post-marketing surveillance and adherence to guidelines [15]. Brieger notes a number of locations where mobile phone interventions have been tried in these areas with examples including SMS–based reporting systems in Uganda, confirming authenticity of drugs in Nigeria, clinical monitoring of patients in Ghana following treatment, and reducing drug stock outs by SMS monitoring of supply in Tanzania [16]. One study in Kenya showed significant success in improved correct management of malaria by healthcare workers who received regular text messages with appropriate case management and motivating quotes [17]. Another application tested in Uganda included using mobile phones to improve diagnostic services by capturing microscopy images and sending them to a central review website for feedback and quality control [18]. This work reports on mobile phone use, which improved detection and treatment of cases in the context of an epidemiological study of malaria in a remote Hill Tract of Bangladesh.

## Methods

This research is part of a larger longitudinal study describing the epidemiology of malaria in Rajbila and Kuhalong Unions of Bandarban Sub District, Bandarban district, Bangladesh [3]. The two unions have an area of about 172 square kilometers and a population of approximately 22,000. There are 4,632 households with about four to five persons in a household and about 50% literacy. A majority of the population are tribal people, the predominant tribe being Marma. A census was carried out initially to define the demographic information of the population; this was updated every four months. The GIS coordinates of each household were also recorded. Malarial infections were documented through both active and passive surveillance. For active surveillance, a random subset of the population, about 40 people a week, was selected for screening regardless of symptoms, using rapid diagnostic tests for both *P. falciparum* and *P. vivax* (Falcivax, Zephyr Biomedicals, Verna, Goa, India) and blood film microscopy. Blood was also collected on filter paper for PCR analysis which was performed at a later time. For passive surveillance, symptomatic persons informed one of the 20 field workers who worked in the communities of their symptoms, or they called a team member (most often the medical officer) to report their symptoms. A field worker then goes to the person’s home to obtain a blood sample to test for malaria using RDT on site, obtain a blood film for microscopy and filter paper for PCR. If the RDT tested at the site of the person’s presentation, or microscopy at the field station was positive, the patients were treated according to national guidelines with artemether-lumefantrine, either immediately in the home for a positive RDT or a day later for negative RDT but positive microscopy (only two cases). PCR was only used for research purposes and not to make clinical decisions. In the beginning of the study, symptomatic persons informed field staff during their frequent visits to communities, and mobile phones were used later as an alternative means of communication when they became available. Specific training on mobile phone use was not given by study personnel.

Participating households in these two unions were provided with phone numbers of the project physician and four other project personnel and informed to call if they had a fever, frequent vomiting, altered mental status, convulsions, yellow discoloration of the eyes or any other serious medical concerns. They were told that the study team could arrange transport if the patient required hospitalization. A strategy was also developed so that people who did not have available credit on their phones (or did not want to use what credit they had), were instructed to call the study physician and then hang up prior to the phone being picked up (“missed call”). The study physician would then call back any missed calls.

The study team began tracking and documenting mobile phone calls in 2010 when an increase in detection of malaria cases using mobile phone calls was first noted. To gain better understanding of the use of mobile phones, an additional study survey on mobile phone use was implemented between July 2011 and July 2012 as part of the regular demographic surveillance with all 4,632 participating households. Questions in this mobile phone survey included number of phones owned, access to borrowed phones, reasons for lack of ownership, history of use of phone for medical purposes or receiving health messages, preference for text *versus* picture messages, and preference for receiving medication reminders and filling out surveys over phone.

Written informed consent was obtained from all household heads as well as individuals from whom blood specimens were obtained who participated in these studies. The study protocol was approved by the Johns Hopkins Bloomberg School of Public Health and the icddr,b Ethical Review Committee.

## Results

**Figure 1:**
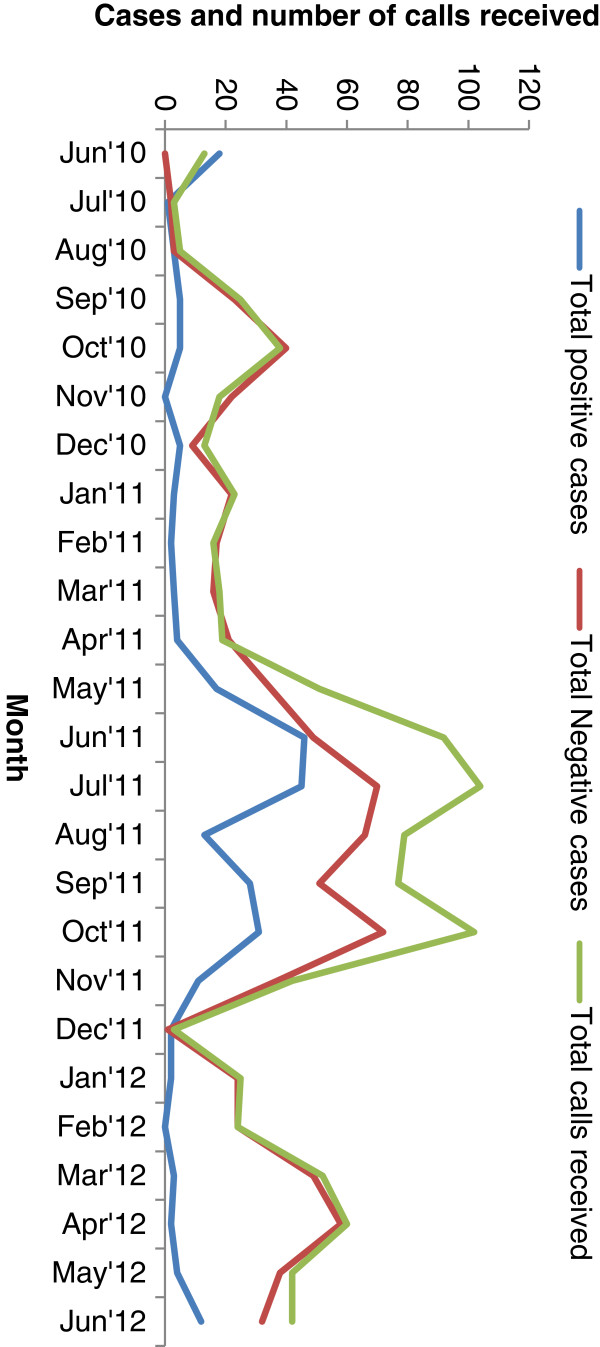
**Cell phone calls and malaria case detection by month.** Temporal pattern of total calls (green line) received by research staff indicating gradual increase in use of cell phones soon after service introduction in the local unions, peaking during peak malaria months yet remaining above 20 calls a month. Of those tested as a result of calls, total positive malaria cases (blue line) cluster in May-October, particularly in 2011, and cases testing negative for malaria (red line) peaking during the same season, but remaining higher for most of the year than the positive cases.

From June 2010 to June 2012, 986 people called the study team to report suspected malaria in one or more people in their household. 857 (87%) of them contacted the medical officer first. Some callers contacted multiple study team members using backup numbers provided and some called multiple numbers or multiple times. These multiple calls from one individual at one time were counted as one call. Those who contacted project staff were then referred to the medical officer for follow up. Sometimes more than one individual was tested in a household or village as a result of one call, resulting in testing of 1,046 people from the 986 individual calls. There were 610 unique numbers out of total 986 mobile calls. Out of 1,046 people tested 265 (25%) positive for malaria by RDT and/or microscopy. The number of mobile phone calls received and of malaria testing and diagnosis in the two study years are presented in Figure 1.

**Figure 2:**
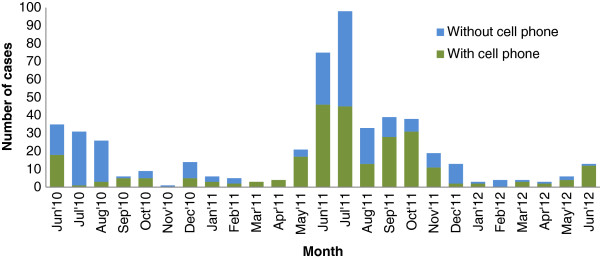
**Symptomatic cases of malaria detected with or without cell phone over time.** The field study’s passive surveillance case detection of symptomatic individuals tested in their home shifted from a minority of case detection initiated by cell phones at the end of 2010 and early 2011 to a majority of detected malaria cases initiated by mobile phone contact by mid 2011 and 2012.

Of a total of 509 symptomatic malaria cases identified from passive surveillance in the two study years, mobile phones were used in 265 (52%) cases. The temporal variation of cases detected with and without mobile phone use from June, 2010 to June, 2012 is presented in Figure 2.

The proportion of symptomatic cases reported by mobile phone was higher in Rajbila than Kuhalong (which is closer to Bandarban town) union, but the difference was not statistically significant. The number of calls from villages with no road access was also compared to those near roads. In this area, 30 villages with a total population of 3,395 (16% of study population) were found to be non-accessible, and 77 villages with a population of 18,338 (84% of study population) were accessible via road. Of a total of 265 cases detected as a result of mobile phone calls, 158 (60%) came from communities with road access, while 107 (40%) from communities without. Of a total of 986 calls, 681 (69%) came from communities that were road accessible and 305 (31%) came from the population that was non-accessible by road. This equates to a significantly higher ratio of positive cases to number of calls (p < 0.001) in the non-accessible areas with 107 cases from 305 calls (35%) compared to 158 cases from 681 calls (23%).

Twelve villages with a total population of about 1900 people (8.6% of study population) in the study area had no network coverage in the villages themselves. From residents of these villages, there were 167 calls (16.9% of calls) that led to malaria testing of 180 people (17.2% of total tested), and 62 of 180 tested (34.4%) were positive. Despite lack of coverage, these communities without a working network were able to reach a mobile phone to call for help as often as those which did have coverage. To make these calls from villages without coverage, persons generally had to climb to the top of a nearby hill to gain access to coverage (about 50 to 150 meters from household).

The results of the mobile phone survey can be found in Table 1. The survey found that of 4,632 households surveyed, 968 (20.9%) owned at least one phone; 126 (2.7%) owned more than one phone; 81(1.7%) reported to have access to borrowing a phone from a friend or neighbor to make a call; 3,583 (77.4%) reported no access to phones. Despite these responses by survey, the study found that of the 194 households that had at least one positive case detected as a result of a cell phone call, 148 (76.3%) had stated they had no access in this survey.

**Table 1:** Cell phone survey results

**Survey question topic**	**Results**
Cell phone ownership (N = 4,632 households)	842 (18.2%): Own 1 phone
	126 (2.7%): Own more than 1 phone
	81 (1.7%): Have access to borrowing a phone (if didn’t own)
	3,583 (77.4%): No access to phones
Reasons for not owning/using phone (N = 3,583)	1782 (49.7%) Never needed to use one
	1449 (40.4%): Cannot afford it
	214 (6.0%): Connection/coverage problems
	138 (3.9%): Don’t know how to use one
Use of phone for medical purposes (N = 968)	546 (56.4%): Yes
	422 (43.6%): No
If yes, for what specific purpose (N = 546)	461(84.4%): Emergency medical care
	65 (11.9%): Malaria services
	20 (3.7%): Other services
If currently receiving health messages (N = 968)	292 (30.2%): Yes
	24 (2.5%): No
	652 (67.4%): Don’t know
Text messaging use (N = 968)	468 (48.3%): Use text messaging (all or some of the time)
	500 (51.7%): Never use text messaging
If use text messaging, preference on type (N = 468)	394 (84.2%): Prefer words and pictures

	73 (15.6%): Prefer words only
	1 (0.2%): Prefer pictures only
If medication reminders would be helpful (N = 968)	675 (69.7%): Yes
	18 (1.9%): No
	275 (28.4%): Don’t know

Survey was conducted between July 2011, and July 2012 among the 4,632 households in the study area.

**Figure 3:**
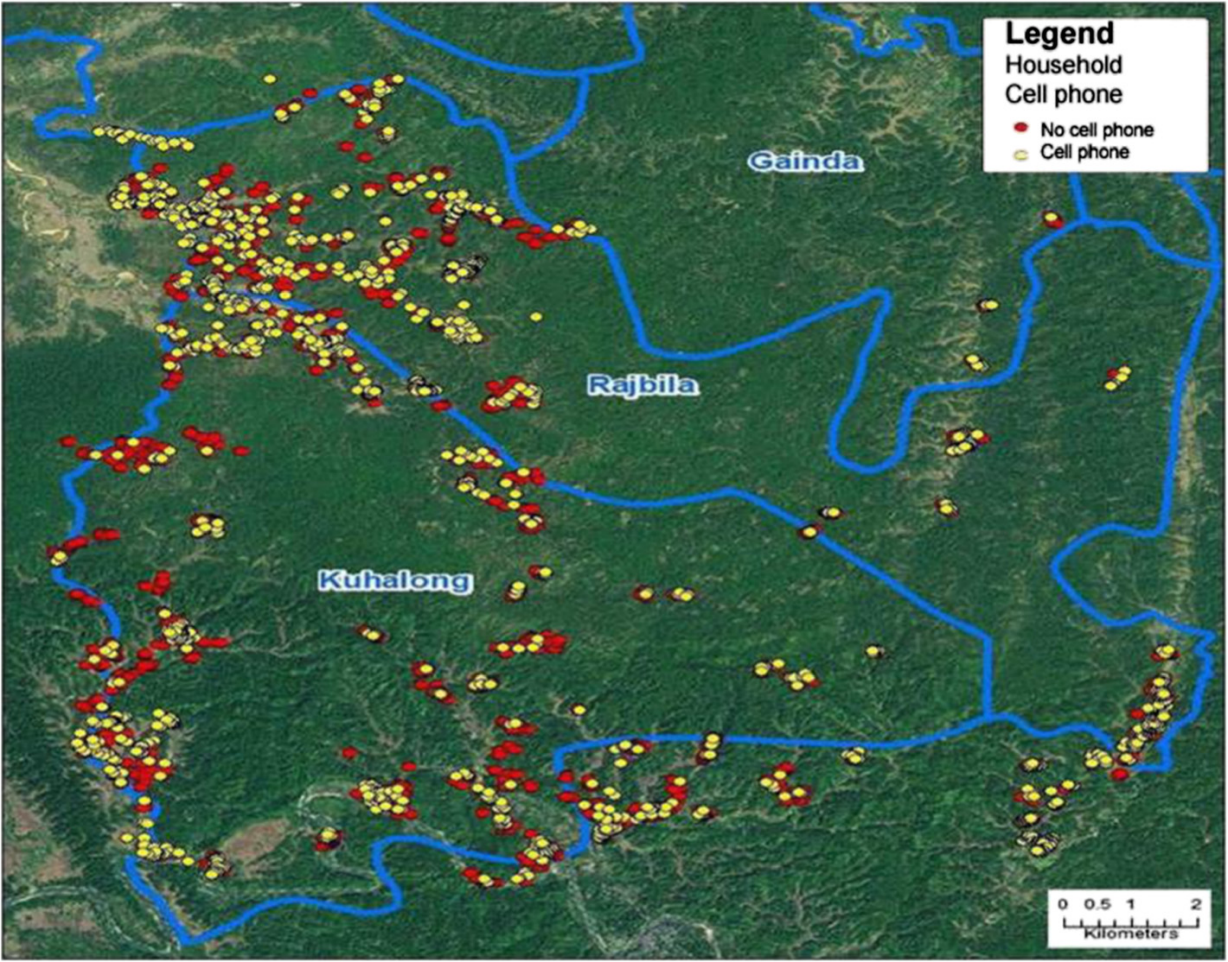
**Cell phone distribution in 2 unions.** Map of mobile phones in households for year 2010 by self-report from mobile phone specific survey initiated as an additional project. 1401 households in yellow have mobile phones and 3239 in red were without mobile phones. The distribution was widespread with all but a few household clusters with nearby access to mobile phones.

Figure 3 shows the distribution of the households who owned or did not own a mobile phone by self-report on a study questionnaire in 2010. Of note, most communities had at least some cell phone owners. For those who did not have a mobile phone, the major reasons for not owning or using one were that they never needed to use one (49.7%), cost (40.4%), connection or coverage problems (6.0%), and not knowing how to use one (3.9%).

For those who did own a phone, just over half (56.4%) stated they had used their phones for medical purposes before, most (84.4%) of these for emergency medical care, and a small number (11.9%) for malaria services. Of current mobile phone owners, 30.2% said they were currently receiving health messages related to National Immunization Days or other public health topics.

With respect to how phones are used, 48.3% of current mobile phone owners said they sometimes use text messaging. Of this group, 84.2% said they preferred text messages with words and pictures, while 15.6% preferred words only. Only one person (0.2%) preferred just pictures. Most (69.7%) stated they would find reminders to take medications helpful, with 28.4% stating they did not know. The majority (67.6%) said they would be willing to respond to surveys over the phone.

## Discussion

This study illustrates how mobile phones can be utilized as an effective tool for reporting febrile illness and for requesting assistance for malaria testing and treatment. Mobile phones have expanded rapidly even into some of the most remote areas of Bangladesh. In this study site in two remote unions near Bandarban city within the Chittagong Hill Tracts, almost every community had at least a few members with a mobile phone. Although mobile phone coverage is not complete, people are able to climb to the top of the hills to get a signal. Despite low household phone ownership and access to borrowed phones, many who did not claim to have access were able to find a way to call. It is plausible that families that do not own a mobile phone may make a greater effort to find a mobile phone in the context of a medical emergency. Also, some families may have gained access to mobile phones since the time of survey. As most communities had at least a few mobile phone owners, it would be logical that these may have been found and borrowed in this context.

In this study, the mobile phone use was shown to increase communication between the study team and participants allowing more careful follow up. Not only has the use of mobile phones helped increase more potential cases tested and followed, optimizing the research agenda, but also it provided a great service to the community. With access to mobile phones, community members were able to have access to rapid diagnosis and treatment, possibly reducing the likelihood of reliance on local medicine men or drug vendors, and therefore the risk of incorrect results and inappropriate medical advice/treatment [13]. Thus, use of mobile phones, when combined with a strong national health program that provides correct treatment options, could also reduce the threat of drug resistance [16].

Mobile phones allowed a prompt treatment of patients at home since the call alerted the field workers to visit the patient’s home, leading to an early RDT testing and treatment. This rapid response is especially important for more severe cases who were now able to receive rapid treatment without travelling long distances to reach services. Provision of prompt treatment is likely to improve clinical outcomes. In the future, mobile phones could also be used to provide treatment reminders and follow-up, and current mobile phone owners generally quite open to receiving such reminders. In malaria endemic areas where many are not literate, picture messages could potentially be used for such reminders, but in this survey most preferred a combination of written and picture messages. These strategies have been shown to be successful in robust randomized trials of text messages to improve adherence [19]. A potential constraint to this approach, however, is that many of the phones do not accommodate picture messages.

Although lack of road access did not affect the likelihood of phone use, the proportion of positive cases from these calls was higher from communities without road access and the potential benefit of mobile phone health calls for the communities without road access was likely greater than those with road access in terms of saving time and providing access to services. Furthermore, the ability of the study team to provide these services has improved the quality of communication between communities and the study team staff. An additional benefit to the efficiency of the project is the ability of the research teams to call families prior to routine visits to families living in remote villages to confirm that the family is home.

There are several challenges when using mobile phones: a) When a patient calls in the evening, the patient or field worker may have to wait until the next morning since travel after dark may not be safe; b) Occasionally when responding to a call, the field workers found that the family had already left to seek other help before the team arrived; c) For those without mobile phone coverage, the family member may have climbed to the top of a hill to make the emergency call, but was not able to receive a follow-up call after they returned to their village; d) Sometimes people have little credit on their phone so they cannot speak for a long time. In this case, the missed call mechanism used by the study physician was found to be quite effective; e) Finally, if the physician’s phone is lost or stolen, important phone calls might be missed. This risk was minimized by leaving five different numbers to call with each household.

There are other possible concerns from shared mobile phone use related to stigma and privacy when integrating mobile phones into health programmes in developing countries [20]. Although this study mainly used mobile phones to respond to calls, other studies where text reminders were sent for medication adherence faced difficulties with privacy/confidentiality since these text messages could potentially be seen by other households or community members. In the context of malaria, which has no associated stigma, the advantages of rapid diagnosis and treatment far outweigh these potential concerns. However, if such programmes were expanded to more sensitive issues such as HIV/AIDS or STDs, these concerns would have to be weighed carefully with potential benefits.

Positive impact of mobile phone use in this study is consistent with a few other reports of using mobile phones in malaria programmes, especially to encourage the use of appropriate guidelines by healthcare workers. A study in Kenya showed that health worker adherence to treatment guidelines for outpatient malaria treatment was improved with the use of text message reminders [21]. One study reported monitoring anti-malarial drug use and adverse reactions in Nigeria [11]. Another study in Thailand used mobile phones for case investigation, follow-up for treatment compliance and monitoring patients’ symptoms [13].

A limitation of this research study is its generalizability. This project field site is one which is intensely monitored by the study’s field staff. The field workers were recruited from the project area so they have detailed knowledge of the area and the people. The health care staff serving other areas may not be as familiar with their catchment area. The key to successful use of mobile phones in similar programmes will depend not only on the mobile phone technology, but on pairing the technology with the on-the-ground knowledge of the area and the people. Nevertheless, this study has demonstrated an effective means for covering remote areas and using mobile phones as a tool to improve project efficiency, as well as quality and timeliness of care as non-governmental organizations and government agencies expand their programmes. As the use of mobile phones is dramatically increasing, the way in which mobile phones are used will continue to change and expand.

## Conclusion

Mobile phones may emerge as a vital tool in case detection and delivery of healthcare services in the Chittagong Hill Tracts, Bangladesh. Mobile phone networks were acquired only recently in this area of the country, almost a decade later than the rest of Bangladesh. The study findings show that many cases of malaria were detected and treated appropriately, even in remote areas, with the help of mobile phones. This suggests that mobile phones may serve as an integral tool in malaria case detection and have potential for becoming an important tool for broader research and health service delivery programmes. The study findings suggest that many cases of malaria can be detected and treated appropriately, even in remote areas, with the help of mobile phones. This type of intervention could easily be adopted as part of public health interventions, such as malaria control programmes implemented by the National Malaria Control Programme of Bangladesh, to improve access, reporting and implementation efficiency. This may prove to be especially beneficial to the populations residing in remote areas without easy access to health care services.

## Competing interests

The authors declare that they have no competing interests.

## Authors’ contributions

CP, JK, LJE, SA, MR, MMN, DAS, DJS, WAK conceived and designed the study. TS, GEG helped in GIS mapping with Assistance of SA, MSH, MR, DAS, DJS, WAK. MSH, MR, CP, KLS, LJE, DAS, DJS, WAK analyzed the data. CP, KLS, LJE, MMN, DAS, DJS, WAK wrote the paper. All authors read and approved the final manuscript.
